# LXR-Mediated Inhibition of CD4^+^ T Helper Cells

**DOI:** 10.1371/journal.pone.0046615

**Published:** 2012-09-28

**Authors:** Laura A. Solt, Theodore M. Kamenecka, Thomas P. Burris

**Affiliations:** Department of Molecular Therapeutics, The Scripps Research Institute, Jupiter, Florida, United States of America; Baylor College of Medicine, United States of America

## Abstract

T_H_17 cells, which require the expression of both retinoic acid receptor-related orphan receptors α and γt (RORαand RORγt) for full differentiation and function, have been implicated as major effectors in the pathogenesis of inflammatory and autoimmune diseases. We recently demonstrated that the Liver X Receptor (LXR) agonist, T0901317 (T09), also displays high-affinity RORα and RORγ inverse activity, potentially explaining its effectiveness in various T_H_17-mediated autoimmune disease models. However, recent studies suggest that in conjunction with the RORs, LXR mediates a negative regulatory effect on T_H_17 cell differentiation. Since T09 acts on both LXRs and RORs, it presents as a valuable tool to understand how compounds with mixed pharmacology affect potential pathological cell types. Therefore, using T09, we investigated the mechanism by which the LXRs and RORs affect T_H_17 cell differentiation and function. Here we demonstrate that T09 activity at RORα and γ, not LXR, is facilitating the inhibition of T_H_17 cell differentiation and function. We also demonstrate that LXR activity inhibits the differentiation and function of T_H_1, T_H_2 and iT_reg_ cells. Finally, T09 inhibited T cell proliferation and induced cell death. These data help explain much of the efficacy of T09 in inflammatory models and suggest that the generation of synthetic ligands with graded, combined LXR and ROR activity may hold utility in the treatment of inflammatory and autoimmune diseases where targeting both T_H_17 and T_H_1 cells is required.

## Introduction

T_H_17 cells are a subset of CD4^+^ T cells characterized by the secretion of IL-17A, IL-17F, IL-21, and IL-22, and play an important role in the regulation of immune responses against bacterial and fungal infections [1], [2], [3]. However, these cells have been linked to the pathology of many autoimmune diseases, including multiple sclerosis, psoriasis, rheumatoid arthritis, and inflammatory bowel disease [3], [4]. Characterization of the development and function of T_H_17 cells has revealed an indispensable role for the orphan nuclear receptors (NR), RORγt and RORα [5], [6]. Mice deficient in RORαand RORγ are defective in the production of IL-17A and IL-17F and are resistant to experimental autoimmune encephalomyelitis (EAE), a mouse model for multiple sclerosis [5], [6]. Therefore, identifying and developing novel ROR-specific inhibitors that can deter T_H_17 cell differentiation and/or function is a valid strategy for designing therapeutics aimed at treating T_H_17 mediated disorders.

We initially characterized the benzenesulfonamide liver X receptor (LXR) agonist T0901317 (T09) as a promiscuous ligand that modulates the activity of several NRs [7]. T09 represented the first identified high-affinity synthetic ligand that targets both RORα and RORγ. However, its promiscuous activity limited utilization of this compound as a tool to examine the function of the RORs. Therefore, using the benzenesulfonamide T09 scaffold as a lead compound, we developed a derivative, SR1001 that was devoid of all LXR activity yet retained its ability to suppress the activity of RORα and RORγ. SR1001 was effective at suppressing T_H_17 cell differentiation and function *in vitro* and *in vivo* and delayed the onset and severity of EAE [8]. However, SR1001 was not as effective as T09 at inhibiting the onset and severity of EAE, suggesting that the mixed pharmacology of T09 was potentially affecting more than just T_H_17 cell differentiation and function[9], [10].

Factors other than T_H_17 cells influence the pathology of inflammatory autoimmune diseases, including EAE. Until recently, the primary effector T cells thought to mediate the pathology of both EAE and multiple sclerosis were the CD4^+^ T helper 1 cells (T_H_1). T_H_1 cells are commonly present along with T_H_17 cells in inflamed tissues. Adoptive transfer experiments of myelin-specific CD4^+^ T_H_1 cells into naïve mice induced EAE [11], [12], [13], [14], [15], [16], [17]. Increased clinical activity in MS correlated with the expression of INFγ and IL-12 in the CNS and CFS [18]. Additionally, STAT4 and T-bet, transcription factors critical for T_H_1 differentiation, are essential for EAE induction [16], [19], [20], [21]. However, deletion of INFγ, the main effector cytokine of T_H_1 cells, rendered mice more susceptible to EAE, suggesting that the differentiation pathway that generates T_H_1 cells was critical for EAE, but not T_H_1 effector cytokines [22], [23]. Therefore, T_H_1 cells are sufficient to cause EAE and play a role in the pathology of the disease, but are not the only cells responsible for disease progression. Thus, the identification of pathological T_H_17 cells as mediators of several autoimmune diseases caused a great deal of excitement leading to an enormous amount of research characterizing the function of these cells.

Several studies have evaluated the effects of LXR activity on EAE disease induction and progression using the LXR agonists T09 and GW3965 [9], [10]. These studies have demonstrated the efficacy of LXR agonists in delaying the onset, severity, and progression of disease in an established model of EAE [9], [10]. These studies highlighted the inhibition of T_H_17 cell differentiation and function in EAE and suggested that the effects on T_H_17 cells was due to LXR activity [9], [10]. Given this data, we returned to our lead compound, T09, to further investigate its effects at inhibiting T_H_17 cells. In the present study, we demonstrate that T09 not only inhibits T_H_17 cell differentiation and function, it also inhibits the differentiation and function of all of the CD4^+^ T helper cell lineages, including T_H_1, T_H_2, and inducible T regulatory cells (iT_reg_). Furthermore, we discovered that the T09-mediated LXR activity decreased CD4^+^ T cell proliferation and led to cell death. Blocking LXR with a LXR antagonist rescued the T09-mediated cell death, but not the inhibition of IL-17. Our studies suggest that T09 inhibits T_H_17 cell differentiation and function through both the RORs and LXRs, whereas LXR activity alone is responsible for the inhibition of the other CD4^+^ T cell subsets. While total inhibition of CD4^+^ T helper cells is not optimal for the development of therapeutics, our data sheds light on the utility of developing therapeutics with less activity at LXR while retaining ROR activity for the treatment of autoimmune diseases. These therapeutics would still specifically target T_H_17 cells, yet also inhibit the T_H_1 arm of autoimmune disease, presenting the possibility of a superior therapeutic for the treatment of autoimmune diseases associated with aberrant T_H_1 and T_H_17 activity.

## Materials and Methods

### Mice

C57BL/6 mice (8 weeks old) were purchased from Jackson Laboratory (Bar Harbor, ME). All mice were maintained and experiments were performed in specific pathogen free environments in accordance with protocols approved by the Institutional Animal Care and Use Committee.

### Cell lines

PBMCs were obtained from Astarte Biologics and maintained in RPMI-1640 with 10% FBS and antibiotics (penicillin and streptomycin; Invitrogen).

### Chemicals

T0901317 (T09) was purchased from Cayman Chemical Company. GW3965 was purchased from Sigma. SR1001 was synthesized as previously described [8]. GSK2033, was synthesized as previously described [24].

### Analysis of Splenocytes

Splenocytes were isolated from spleen as previously described [8]. The conditions for the different T_H_ cell subsets were: 20 µg/mL anti-IL-4 and 20 µg/mL anti-IFNγ for T_H_O (neutral conditions); 20 µg/mL anti-IL-4, 20 ng/mL IL-12 and 10 ng/mL IFNγ for T_H_1 conditions; 20 µg/mL anti-IFNγ and 10 ng/mL IL-4 for T_H_2 conditions; 10 µg/mL anti-IFNγ, 10 µg/mL anti-IL-4, and 2 ng/mL TGFβ for iT_reg_ conditions; 20 µg ml^−1^ anti-IFNγ, 20 µg/mL anti-IL-4, 1 ng/mL TGFβ, and 10 ng/mL IL-6 for T_H_17 conditions. All cultures were stimulated with 1 µg/mL anti-CD3 and 1 µg/mL anti-CD28. Four to five days after activation, all cells were restimulated with 5 ng/mL phorbol-12-myristate-13-acetate (PMA) (Sigma) and 500 ng/mL ionomycin (Sigma) for 2 hours with the addition of GolgiStop (BD Bioscience) for an additional 2 hours before intracellular staining. Similar restimulation with PMA and ionomycin occurred for ELISA and hPBMCs. Intracellular cytokine staining was performed using BD Biosciences Cytofix/Cytoperm kit according to manufacturer's instructions. Propidium iodide staining was performed in accordance to manufacturer's instructions (BD Bioscience). CFSE labeling (Invitrogen) was performed in accordance with the manufacturer's instructions. Cells were cultured in RPMI 1640 medium (Invitrogen) with 10% FBS and antibiotics. Flow cytometric analysis was performed on a BD LSRII (BD Biosciences) instrument and analyzed using FlowJo software (TreeStar). FACS analysis was performed using antibodies specific to CD4, IL-17, IFNγ, Foxp3, and IL-4.

### ELISA

Concentration of IL-17A and IL-17A/F in the culture supernatant was determined by ELISA kits according to the manufacturers protocol (R & D Systems).

### MTT assay

Splenocytes were isolated and differentiated under T_H_ polarizing conditions for 3 days. MTT assay was performed using a Vybrant MTT Cell Proliferation Assay Kit (Invitrogen) according to the manufacturer's protocol.

### Reverse transcription (RT) PCR

Splenocyte total RNA was extracted using a RNeasy Plus Micro Kit (Qiagen) and reverse transcribed using iScript cDNA biosynthesis kit (Bio-Rad). Quantitative RT-PCR was performed with a 7900HT Fast Real Time PCR System (Applied Biosystems) using SYBR Green (Roche) as previously described [25]. Primer sequences for mouse IL-17A, IL-17F. IL-21, IL-22, IL-23R, ABCA1, ABCG1, GATA3, T-bet, IL-4, and Foxp3 have been previously described [5], [26], [27], [28]. The level of mRNA expression was normalized to that of GAPDH mRNA expression.

### Cell death analysis

Splenocytes were cultured as previously described [8]. The cells undergoing apoptosis were detected by FACS using FITC-Annexin-V apoptosis detection kit (BD Bioscience).

### Statistical analysis

All data are expressed as the mean ± s.e.m. (*n = 3* or more). Statistical analysis was performed using unpaired Student's *t*-test. *p* values ≤0.05 are statistically significant and indicated by an asterisk (*).

## Results

### T09 inhibits T_H_17 cell differentiation

Previously, we established that T09 can directly modulate the activity of both RORα and RORγt [7]. We demonstrated that T09 induced a conformational change in both RORα and RORγ that inhibited the interaction of the coactivator SRC-2 leading to reduced activity of each receptor. Furthermore, we demonstrated T09's efficacy at inhibiting IL-17 transcription using an IL-17 promoter driven luciferase reporter[7].

**Figure 1:**
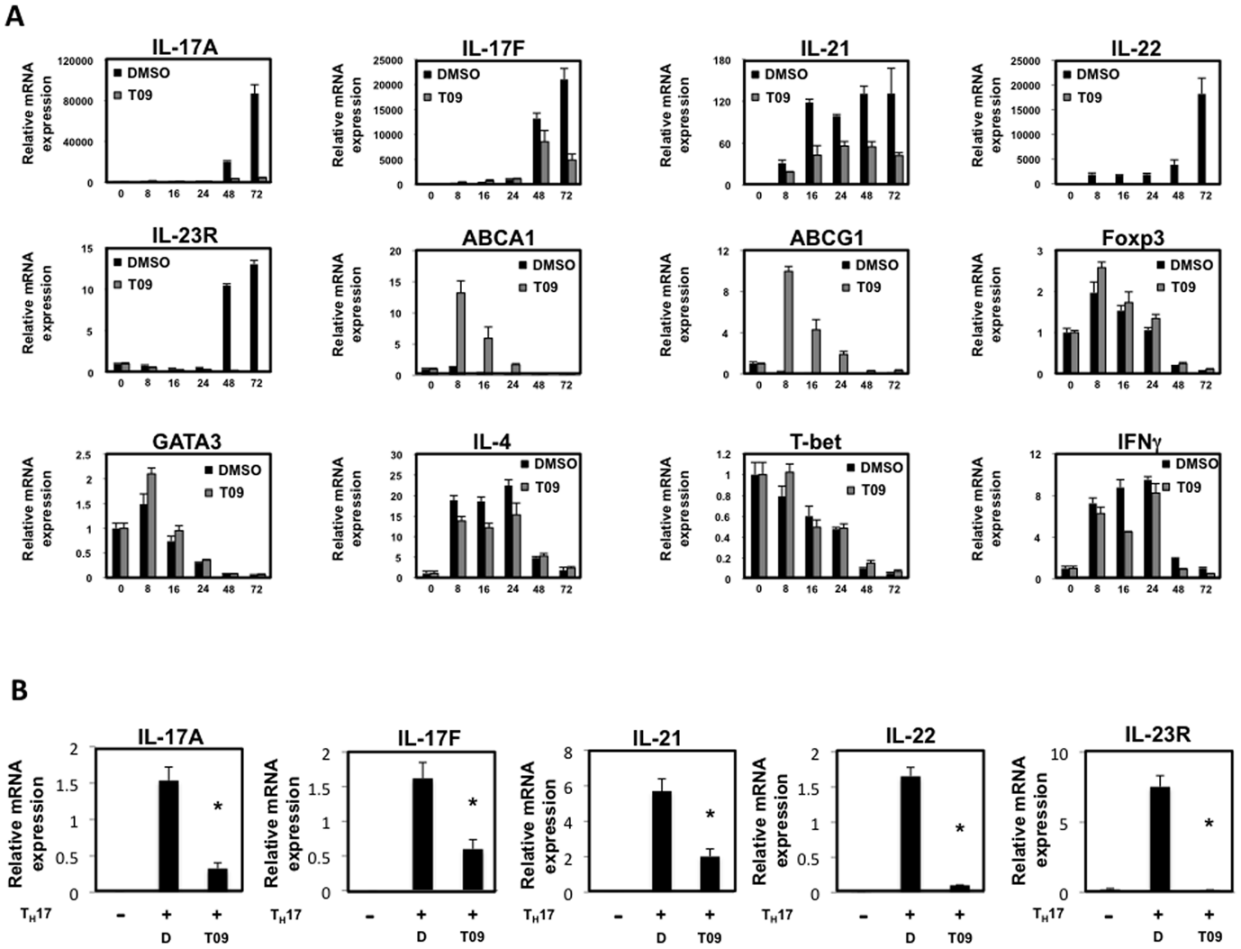
T09 inhibits T_H_17 cells cytokine expression. *A*, Realtime - RT PCR analysis of splenocytes cultured under T_H_17 polarizing conditions in the presence of T09 (5 µM) for the duration of the time course. (Data are normalized to GAPDH). *B*, Realtime - RT PCR analysis of splenocytes cultured under T_H_17 polarizing conditions for a total of three days (72 hours). T09 (5 µM) was added into the cultures after day 2 (after 48 hours). (Data are normalized to GAPDH). (*n = 3*, * p<0.05).

Given this data, we wanted to further explore the effects of T09 on T_H_17 cell differentiation and function. Therefore, we cultured murine splenocytes under T_H_17 polarizing conditions (TGFβ and IL-6) with T09 (5 µM) or vehicle control for 3 days. The combination of TGFβ and IL-6 increased the mRNA expression of IL-17A, IL-17F, IL-21, IL-22, and IL-23R in vehicle treated cells whereas T09 treated cells failed to significantly upregulate these T_H_17 mediated cytokines and cytokine receptors when evaluated by real-time PCR (RTPCR) (Fig. 1A). However, T09 treatment did upregulate the mRNA expression of the LXR target genes ABCA1 and ABCG1, demonstrating that LXR was active in these cultures (Fig. 1A)[29]. Given that the spleen contains other cell types besides CD4^+^ T cells, including macrophages and dendritic cells, and that LXR activity in macrophages and dendritic cells results in clearance of apoptotic cells through Mertk (Mer receptor tyrosine kinase), we speculated whether the decrease in T_H_17 cell differentiation was due to Mertk activity[30]. An increase in Mertk would decrease IL-23 expression, a cytokine necessary for the propagation and pathogenicity of T_H_17 cells [31]. RTPCR analysis indicated that there was no change in Mertk gene expression in T09 treated T_H_17 splenocyte cultures relative to vehicle control. Interestingly, T09 treatment significantly increased the expression of IL-23 in T_H_17 cultures. However, since the expression of the IL-23R decreased with T09 treatment, the increased IL-23 in cultures was not sufficient to propagate the T_H_17 cells (Figure S1).

Since the T09 treated splenocytes failed to differentiate into T_H_17 cells, we investigated whether the cells were differentiating into any of the other CD4^+^ T helper lineage. Given that TGFβ is critical for the differentiation of both T_H_17 and iT_reg_ cells, we questioned whether the splenocytes were differentiating into iT_reg_ cells [32]. RTPCR analysis confirmed that the cells were not differentiating into iT_reg_ cells since the expression of Foxp3 was similar in both T09 and vehicle treated cultures. Furthermore, the splenocytes did not differentiate into T_H_1 or T_H_2 cells since the expression of the T-bet and GATA3, transcription factors required for T_H_1 and T_H_2 cell development, respectively, did not change relative to vehicle control. Consistent with these data, the cytokines associated with T_H_1 and T_H_2 cells, INFγ and IL-4, did not change as a consequence of T09 treatment relative to vehicle control (Fig. 1A).

Finally, we evaluated whether T09 could inhibit T_H_17-mediated gene expression in cells that had already started to differentiate into T_H_17 cells. Therefore, murine splenocytes were cultured under T_H_17 polarizing conditions for a total of 3 days. Vehicle or T09 (5 µM) was added to the culture twenty-four hours prior to harvest. RTPCR analysis indicated that T09 can inhibit the expression of IL-17A, IL-17F, IL-21, IL-22, and IL-23R, suggesting that T09 can effectively suppress the expression of T_H_17 derived cytokines once the cells have started to differentiate (Fig. 1B). To summarize, these data establish that T09 suppresses T_H_17-dependent gene expression *in vitro*.

### T09 inhibits IL-17 protein production

**Figure 2:**
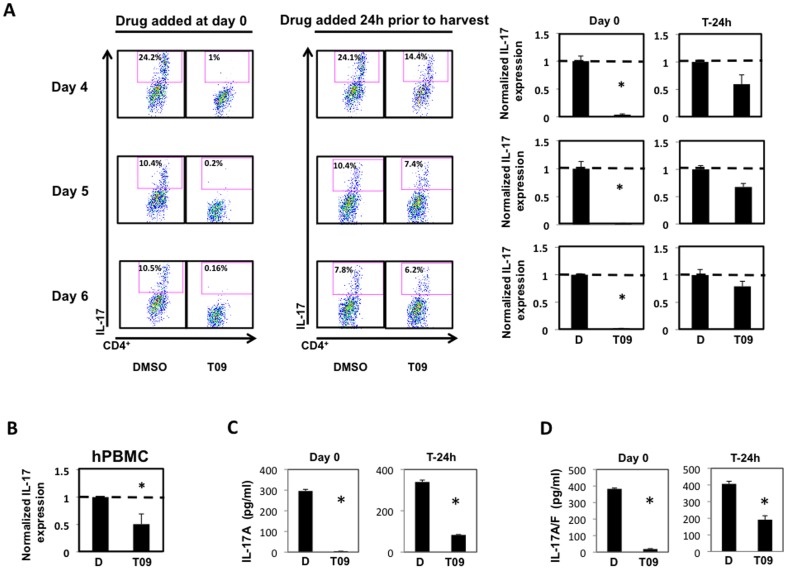
T09 inhibits IL-17 cytokine production. *A*, The effect of T09 (5 µM) on IL-17 cytokine expression during murine T_H_17 cell differentiation. Left: Intracellular cytokine staining on splenocytes treated with T09 for the duration of the time course (4 days). Middle: Intracellular cytokine staining on splenocytes treated with T09 24 hours prior to the end of the time course. Right: Graphs summarizing the FACS plots. Data was normalized to DMSO controls. *B*, The effect of T09 treatment (5 µM, 24 hours) on IL-17 cytokine expression on differentiated human peripheral blood mononuclear cells (hPBMCs) using intracellular cytokine staining. *C*, The effect of T09 (5 µM) on IL-17 cytokine secretion during murine T_H_17 cell differentiation. ELISA assay on splenocytes treated with T09 for the duration of the three day time course (T = 0, left) or treated with T09 24 hours prior to the end of the time course (T-24h, right). *D*, The effect of T09 (5 µM) on IL-17A/F cytokine secretion during murine T_H_17 cell differentiation. ELISA assay on splenocytes treated with T09 for the duration of the three day time course (T = 0, left), or on splenocytes treated with T09 24 hours prior to the end of the time course (T-24h, right). (*n = 3*, * p<0.05).

Because T09 was effective at inhibiting T_H_17-mediated cytokine gene expression, we wanted to establish how this correlated to IL-17 protein expression. Splenocytes were cultured under T_H_17 polarizing conditions for 4, 5, and 6 days, and analyzed for IL-17A expression by intracellular flow cytometry. Consistent with the mRNA data, T09 (5 µM) significantly inhibited IL-17A expression from CD4^+^ T cells when added to the culture at Day 0. Moreover, T09 treatment twenty-four hours prior to harvest inhibited IL-17A expression from CD4^+^ T cells (Fig. 2A). While this effect was not statistically significant, it demonstrates a trend, which is consistent with the mRNA data. Finally, T09 (5M) was also effective at inhibiting IL-17A expression in human peripheral blood mononuclear cells, as evidenced by the decrease in IL-17A expression analyzed by intracellular flow cytometry (Fig. 2B).

In addition to IL-17A, T_H_17 cells express and secrete other soluble factors, including IL-17F[3]. Therefore, we assessed whether T09 treatment would inhibit the cytokine production and secretion of IL-17A and IL-17F. Splenocytes were cultured under T_H_17 polarizing conditions for three days with vehicle or T09 (5 µM) added into the cultures at Day 0 or Day 2 (T-24). Supernatants were harvested from the cultures after three days and evaluated for the presence of IL-17A and IL-17A/F by ELISA. Consistent with the RTPCR data and FACS data, the ELISA assays indicated that T09 inhibited IL-17A and IL-17F secretion over a three-day time course (Fig. 2C and 2D).

### T09 inhibits the differentiation of T_H_1, T_H_2, and iT_reg_ cell lineages

**Figure 3:**
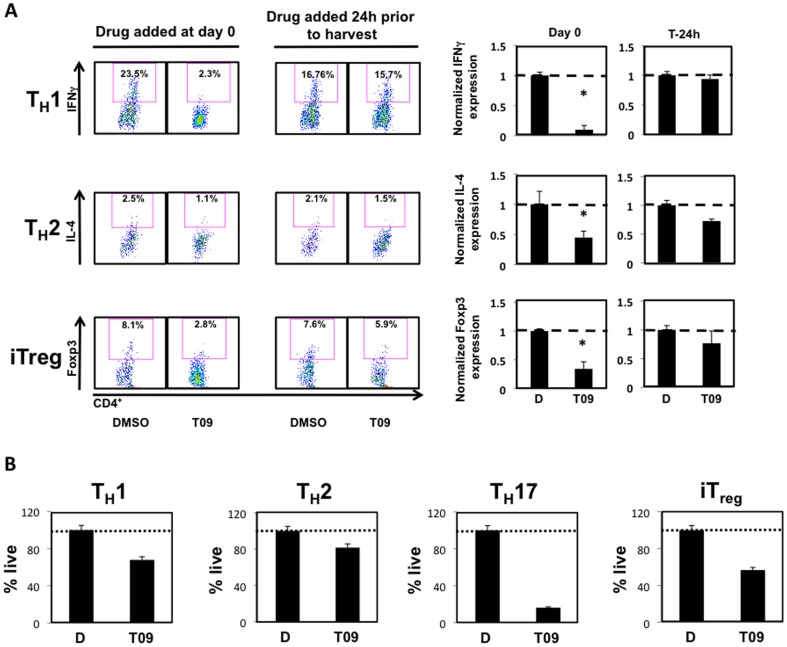
T09 inhibition of the differentiation and effector function of T_H_1, T_H_2, and iT_reg_ cells is a consequence of cell death. *A*, The effect of T09 (5 µM) on murine T_H_1, T_H_2, and iT_reg_ differentiation. Left: Intracellular cytokine staining on splenocytes treated with T09 for the duration of the time course. Middle: Intracellular cytokine staining on splenocytes treated with T09 24 hours prior to the end of the time course. Right: Graphs summarizing the FACS plots. Data was normalized to DMSO controls. *B*, The effect of T09 (5 µM) on the viability of T_H_1, T_H_2, T_H_17, and iT_reg_ cells. Cells were FASC analyzed and gated on Annexin V and PI negative cells and normalized to DMSO controls. (*n = 3*, * p<0.05).

Our data suggests that T09 is an effective inhibitor of T_H_17 cell differentiation and function. Given that T09 is such a potent inhibitor of disease onset and progression in several models of autoimmune disease, we questioned whether T09 inhibited the differentiation and function of other CD4^+^ T cell subsets, specifically T_H_1, T_H_2, and iT_reg_ cells[9], [10]. Splenocytes were cultured under T_H_1, T_H_2, or iT_reg_ differentiating conditions for a total of 5 days. T09 (5 µM) was added to the culture media at either the initiation of the experiment (Day 0) or after 4 days (24 hours prior to harvest) and analyzed by intracellular flow cytometry for the expression of IFNγ, IL-4, or Foxp3 (Fig. 3A). Surprisingly, T09 significantly inhibited the expression of IFNγ, IL-4, and Foxp3, when added at the initiation of the experiment (Fig. 3A, *left FACS panels*). Furthermore, addition of T09 twenty-four hours prior to cell harvest had a small inhibitory effect on the expression of IFNγ, IL-4, and Foxp3 (Fig. 3B, *right FACS panels*). While this effect was not statistically significant, it demonstrates a trend, which is consistent with the data seen in Figure 2. Due to T09's inhibition of all CD4^+^ T cell populations, we analyzed T_H_1, T_H_2, T_H_17, and iT_reg_ cultures for cell viability using AnnexinV and propidium iodide staining. FACS analysis indicated that T09 was inducing cell death since the number of viable cells in each of the splenocyte cultures was less than vehicle control (Fig. 3B). Collectively, this data suggest that T09 not only inhibits the differentiation and function of T_H_17 cells, but also inhibits the differentiation and function of T_H_1, T_H_2, and iT_reg_ cells.

### LXR activation inhibits T helper cell differentiation and cell proliferation

**Figure 4:**
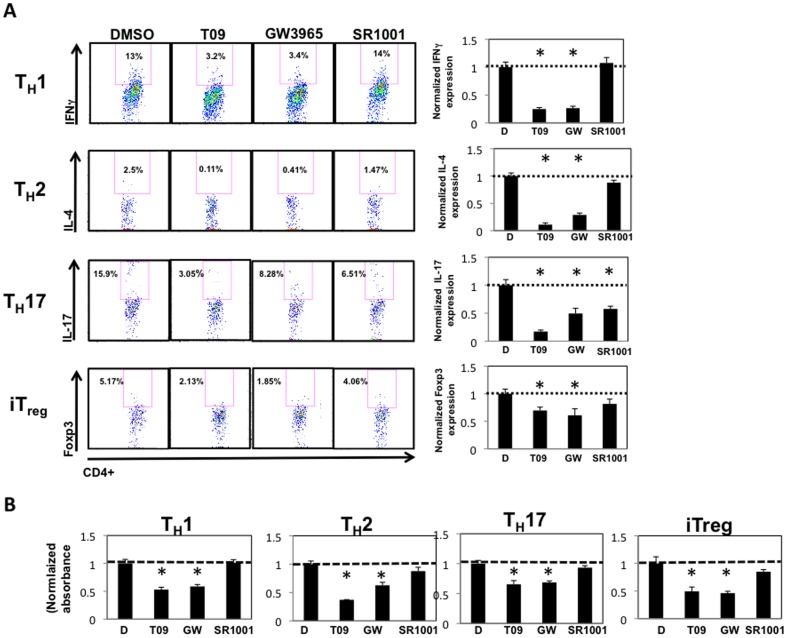
Inhibition of all CD4^+^ T helper lineages is a function of LXR activity. *A*, Intracellular cytokine staining on splenocytes differentiated under T_H_1, T_H_2, T_H_17, or iT_reg_ conditions and treated with vehicle (DMSO), T09, GW3965, or SR1001 (5 µM each) for the duration of the time course (4 days). Right: Graphs summarizing the FACS plots. Data was normalized to DMSO controls. *B*, MTT assays of T_H_1, T_H_2, T_H_17, or iT_reg_ cells cultured with vehicle (D), T09, GW3965, or SR1001 (5 µM each) for the duration of the time course (4 days). Data was normalized to DMSO controls. (*n = 4*, * p<0.05).

We were intrigued that T09 inhibited the differentiation and function of T_H_1, T_H_2, iT_reg_, and T_H_17 cells. However, it was possible that these effects were not mediated by increased LXR activity but rather a consequence of compound toxicity. To address this, we compared the effects of T09 to those of a second LXR agonist, GW3965, as well as SR1001, our recently identified RORα/γ-specific inverse agonist, which is also an analog of T09[8], [33]. Similar to Figure 3, splenocytes were cultured under T_H_1, T_H_2, T_H_17, or iT_reg_ differentiation conditions for a total of 5 days in the presence of vehicle control, T09 (5 µM), GW3965 (5 µM), or SR1001 (5 µM). On day 5, cells were analyzed by intracellular flow cytometry (FACS) for expression of IFNγ, IL-4, IL-17, or Foxp3. Similar to T09, GW3965 significantly inhibited the differentiation and function of T_H_1, T_H_2, T_H_17, and iT_reg_ cells, whereas SR1001 only inhibited the differentiation and function of T_H_17 cells (Fig. 4A).

Our data suggest that inhibition of CD4^+^ T cell differentiation is mediated through LXR, which is consistent with that described by Bensinger *et al* [34]. This group demonstrated that LXR activity modulates cell proliferation and immunity via the reciprocal regulation between the LXR and SREBP transcriptional pathways. Given that LXR and SREBP are key mediators of cholesterol synthesis and cholesterol is a key component of cell membranes, tight control of LXR activity is essential for the proliferation and clonal expansion of T cells[34]. Therefore, dual activation of LXR and SREBP inhibits T cell proliferation [34]. Furthermore, in the absence of sufficient growth signals, T cells fail to proliferate and undergo apoptosis [35], [36]. Therefore, we hypothesized that T09-mediated activation of LXR was inhibiting cell proliferation and leading to cell death in CD4^+^ T cells. To test this hypothesis, we performed MTT assays on T_H_1, T_H_2, T_H_17, and iT_reg_ populations treated with vehicle control, T09, GW3965, or SR1001 for three days. MTT assays are colorimetric assays that assess the viability and proliferation of cells. MTT assays on splenocyte cultures determined that cells treated with T09 or GW3965 failed to proliferate at the same rate as vehicle or SR1001 treated cells (Fig. 4B). These data suggest that LXR activity inhibits CD4^+^ T cell proliferation and ultimately leads to cell death.

### LXR activity regulates T cell proliferation

**Figure 5:**
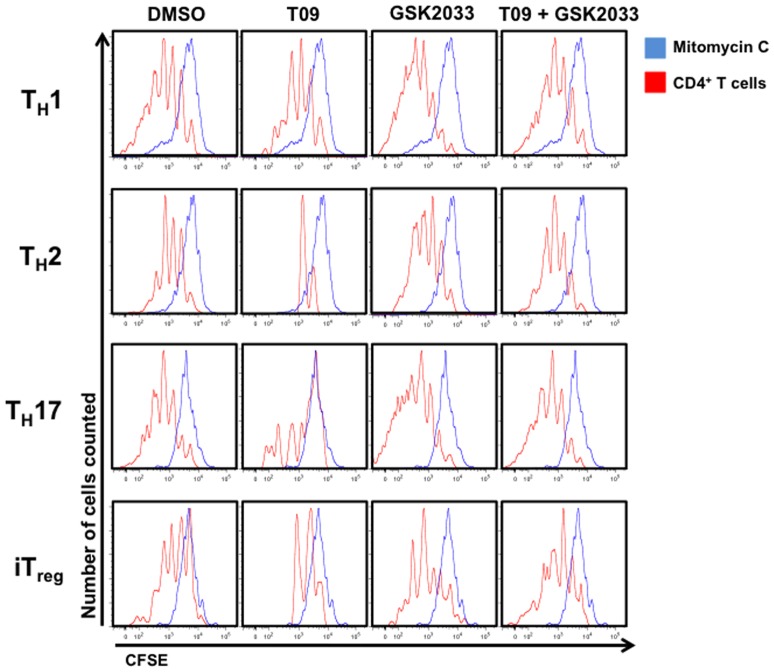
LXR regulates T cell proliferation. CFSE labeled splenocytes differentiated under T_H_1, T_H_2, T_H_17, or iT_reg_ conditions and treated with vehicle (DMSO), T09 (5 µM), GSK2033 (5 µM), or a combination of T09 and GSK2033 (5 µM each) for the duration of the time course (5 days). Cells were FACS analyzed and gated on live, CD4^+^ T cells. (*n = 4*).

To further assess whether LXR activity was regulating CD4^+^ T cell proliferation and cell death, we hypothesized that an antagonist to LXR would increase cell proliferation, and when used in conjunction with T09, would block or “rescue” the effects of T09. To address this, we used a recently described synthetic LXR antagonist, GSK2033, to assess the effects of LXR antagonism on CD4^+^ T cell proliferation and cell death[24]. In order to address the effects of LXR activity on CD4^+^ T cell proliferation, we CFSE (carboxyfluorescein diacetate, succinimidyl ester) labeled cells to quantitatively evaluate cell division. Therefore, splenocytes were first labeled with CFSE and then cultured under T_H_1, T_H_2, T_H_17, and iT_reg_ conditions in the presence of vehicle control, T09 (5 µM), GSK2033 (5 µM, LXR antagonist), or a combination of T09 and GSK2033 (5 µM each) for the duration of the experiment (5 days). A portion of the population was arrested at the parent generation using mitomycin C (blue peaks). After 5 days, the cells were FACS analyzed to determine the relative proliferation of each T helper cell lineage relative to the initial parent population. When compared to vehicle control, T09 treated T_H_2, T_H_17, and iT_reg_ cells failed to significantly proliferate. Interestingly, while T09 did inhibit the proliferation of T_H_1 cells, it did not have as profound of an effect on T_H_1 cells as it did on the other T cell lineages (Fig. 5). More importantly, GSK2033 treatment enhanced the proliferation of T_H_1, T_H_2, T_H_17, and iT_reg_ cells *in vitro* and when added into the cultures with T09, GSK2033 blocked the anti-proliferative effect of T09 giving the cells a proliferation profile similar to vehicle control (Fig. 5). When we analyzed the DNA content of the activated CD4^+^ T cell populations, we found that T09 was arresting the cells in the G0/G1 phase with a large portion of the cells considered apoptotic (Fig. S2). This data correlates to that described by Bensinger *et al*. where they ascertain that LXR activation decreases cell cycle progression in activated lymphocytes[34]. Interestingly, a small fraction of T09 treated T_H_1 cells were found in G2/M phase, which could account for the increased proliferation observed relative to the other T09 treated lineages. Cells treated with GSK2033 had a larger percentage of cells in G2/M, potentially explaining the increased proliferation seen in Figure 5. Finally, GSK2033 blocked the T09-mediated cell cycle inhibition, resulting in a profile similar to vehicle treated cells (Fig. S2). These data further demonstrate that LXR activity modulates CD4^+^ T cell proliferation through regulation of the cell cycle.

### LXR is necessary for IFNγ production from T_H_1 cells, but dispensable for IL-17 production in T_H_17 cells

**Figure 6:**
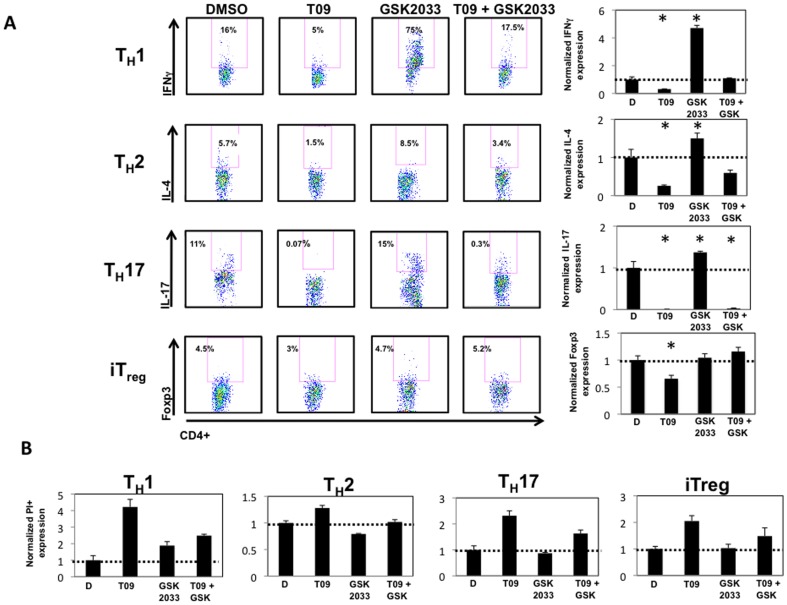
Cell death CD4^+^ T helper lineages is a function of LXR activity. *A*, Intracellular cytokine staining on splenocytes differentiated under T_H_1, T_H_2, T_H_17, or iT_reg_ conditions and treated with vehicle (DMSO), T09 (5 µM), GSK2033 (5 µM), or a combination of T09 and GSK2033 (5 µM each) for the duration of the time course (4 days). Graphs summarizing the FACS plots with data normalized to DMSO controls. *B*, The effect of T09 (5 µM), GSK2033 (5 µM), or a combination of T09 and GSK2033 (5 µM each) on the viability of T_H_1, T_H_2, T_H_17, and iT_reg_ cells. Cells were FACS analyzed and gated on PI positive cells and normalized to DMSO controls. (*n = 4*, * p<0.05).

Given the profound effects of LXR activity on CD4^+^ T cell proliferation, we wanted to verify that lack of proliferation was inhibiting the function of each lineage. Accordingly, splenocytes were cultured under T_H_1, T_H_2, T_H_17, and iT_reg_ conditions in the presence of vehicle control, T09 (5 µM), GSK2033 (5 µM, LXR antagonist), and a combination of T09 and GSK2033 (5 µM each) for the duration of the experiment (5 days)[24]. On day 5, cells were analyzed by intracellular flow cytometry (FACS) for expression of IFNγ, IL-4, IL-17, or Foxp3. Similar to our previous experiments, T09 significantly inhibited the differentiation and function of T_H_1, T_H_2, T_H_17, and iT_reg_ cells. Addition of GSK2033 significantly enhanced the expression of IFNγ, whereas it had a modest effect on IL-4, and IL-17 production with no effect on the expression of Foxp3. However, when GSK2033 was added with T09, it rescued the T09-mediated inhibition of all of the T cells except T_H_17 cells suggesting that T09-mediated IL-17 inhibition is dependent on its activity at RORα and RORγ (Fig. 6A). To determine whether GSK2033 was inhibiting cell death, we propidium iodide stained all cultures after 5 days of drug treatment. FACS analysis indicated that T09 induced cell death, whereas GSK2033 rescued the cell death seen with T09 alone (Fig. 6B). Finally, even at lower concentrations, T09 (3 µM) induces cell death in all CD4^+^ T cell lineages when FACS analyzed for propidium iodide and Annexin V (Fig. S3). Collectively, this data suggests that LXR activity inhibits the differentiation and function of T_H_1, T_H_2, T_H_17, and iT_reg_ cells via regulation of the cell cycle and induces cell death.

## Discussion

The initiation and progression of autoimmune disease is a complex process, one that involves a dynamic interplay between numerous signaling pathways and cell types. The action of one cell type alone is rarely the culprit in the underlying disease pathology. However, identification of cell types that contribute to disease is essential for not only the understanding of the mechanism of disease onset and progression, but for the generation of therapeutics aimed at the treatment of these diseases. The identification of T_H_17 cells as major effectors in the pathology of numerous autoimmune diseases has led to a greater understanding of their development and function[3]. Due to their pathological nature, drug discovery efforts have focused on methods to inhibit T_H_17 cell differentiation and function[8], [37]. Since T_H_17 cell differentiation is dependent on RORα and RORγt activity, and NRs are ligand dependent transcription factors and effective targets for drug discovery, the next logical step was to focus on the development of ligands that would specifically inhibit these two NRs[5], [6]. We originally identified T09 as the first synthetic ROR-ligand as it bound to and altered the activity of RORs[7]. Using T09 as a scaffold, we developed a novel ROR-specific inverse agonist, SR1001, which was effective at inhibiting T_H_17 cell development and function[8]. While this compound was efficacious in a mouse model of EAE, several recent studies have suggested that LXR activity may also negatively regulate T_H_17 cell differentiation and function[9], [10]. Collectively, these studies suggest that the activity of several NRs may affect the outcome of T_H_17 cell differentiation and function. Furthermore, the design of therapeutics with mixed LXR and ROR pharmacology may hold utility in the treatment of autoimmune diseases. T09 presented as a valuable tool to evaluate the effects of both LXR and ROR activity on T_H_17 cell differentiation and function. The current studies were designed to evaluate the consequences of T09 treatment on T_H_17 cells. However, we unexpectedly found that T09 affected the differentiation and function of other CD4^+^ T helper cell lineages.

Our studies demonstrate that T09 suppresses the differentiation and function of T_H_17 cells. This is evidenced by the inhibition of the gene expression of T_H_17-derived cytokines, namely IL-17A, IL-17F, IL21, and IL-22 in splenocyte cultures. T09 also inhibits IL-17 protein production and secretion from T_H_17 cells. Interestingly, T09 increased IL-23 gene expression in T_H_17 cultures. Given that IL-23 plays a critical role in the propagation and maintenance of T_H_17 cells, this increase should help maintain the T_H_17 cell cultures since the IL-23 receptor is usually upregulated on T_H_17 cells as they differentiate, allowing for their expansion [38], [39]. However, this does not appear to be the case since T09 potently inhibited the expression of IL-23R in T_H_17 splenocyte cultures, thus rendering the extra IL-23 ineffective at propagating the T_H_17 cells. These data suggest that both LXR and ROR work together to suppress the development of T_H_17 cells.

Further analysis of T09 treated T_H_17 splenocytes indicated that while the cells did not differentiate into T_H_17 cells, they failed to differentiate into any of the other CD4^+^ T cell lineages, namely T_H_1, T_H_2, and iT_reg_ cells. Furthermore, the induction of the LXR target genes, ABCA1 and ABCG1, proved that LXR was functionally active in the splenocyte cultures and hints at the mechanism by which LXR may be inhibiting T_H_17 cell differentiation. In fact, several groups have established that ABCG1, not ABCA1, regulates cholesterol homeostasis and proliferation in T cells. Since deficiency in ABCG1 leads to enhanced proliferative capabilities, these studies have established that it is the LXR-mediated expression of ABCG1 that causes the anti-proliferative activity observed in T cells [34], [40].

Due to the profound effects T09 was having on T_H_17 cells, we performed more in depth analysis of T09's effect on other CD4^+^ T helper cell lineages. These studies revealed that T09 also inhibited the differentiation and function of T_H_1, T_H_2, and iT_reg_ cells, evidenced by the decrease in expression of their canonical cytokines, IFNγ and IL-4, respectively, or the T_reg_ specific transcription factor, Foxp3. In order to determine whether these effects were LXR or ROR-specific, we used a second LXR agonist, GW3965, as well as a ROR-selective inverse agonist, SR1001, and compared their effects on CD4^+^ T cell differentiation to T09[8], [33]. These studies revealed that the inhibition was LXR mediated as treatment with SR1001 had no effect on the differentiation of any CD4^+^ T helper cell lineage other than T_H_17 cells, whereas similar to T09, GW3965 inhibited the differentiation of all CD4^+^ T cell lineages[8]. Furthermore, the effects observed with both T09 and GW3965 was due to an inhibition of T cell proliferation as MTT assays demonstrated a significant decrease in proliferation in T09 and GW3965 treated cultures whereas SR1001 had no effect. This effect was not due to overt toxicity since lower concentrations of T09 induced cell death as well. Additionally, CFSE labeling revealed that T09 significantly blocked CD4^+^ T cell proliferation occurring via cell cycle regulation. Finally, we demonstrated that T09 was mediating CD4^+^ T cell inhibition and cell death since these effects were rescued when a LXR antagonist, GSK2033, was introduced into the cultures along with T09.

In the present study, we demonstrated that LXR activation, induced by T09 and GW3965, inhibited T cell proliferation and induced cell death. We also demonstrated that inhibition of LXR, using an antagonist, enhances T cell proliferation and when used in conjunction with T09, blocks T cell death. These data are in support of previously published work demonstrating that ligation of LXR during T cell activation inhibits mitogen-driven expansion[34]. Furthermore, it has been established that genetic deletion of LXRβ in T cells confers a proliferation advantage, which is consistent with our data obtained with GSK2033[34]. The anti-proliferative effect and cell death caused by T09 treatment can be attributed to LXR's role in cholesterol metabolism. The maintenance of cellular cholesterol levels during T cell proliferation is a tightly regulated process. When LXR is activated in T cells, the cholesterol efflux transporters, ABCA1 and ABCG1 are upregulated on the surface of activated T cells, thus limiting the cellular cholesterol pools, which are necessary for cell membrane expansion [34], [40]. T cells that are activated but cannot expand due to insufficient energy resources are going to die due by apoptosis[35], [36]. In support of this, apoptosis induced by oxysterols, which are potent LXR agonists, has been described in murine lymphoma cells and normal thymocytes, thus linking LXR activation and T cell apoptosis [41], [42]. Additionally, the LXR mediated T cell death is intriguing given that SPα, a LXR target gene involved in the regulation of apoptosis, has been shown to be upregulated in macrophages, protecting them from cell death[43], [44]. However, since SPα is a LXRα target gene and T cells do not express LXRα, the antiapoptotic effects of LXR activation may be limited to macrophages[34], [43]. Despite these observations, T09 has been used *in vivo* with no obvious observations of T cell apoptosis suggesting that massive LXR activity is not toxic. However, the apoptosis observed in cell culture may be due to limited available growth resources. In this regard, T09 may still be inhibiting cell proliferation, which could account for the *in vivo* efficacy of T09. Despite the recent advances in understanding the links between LXR activity and innate and adaptive immunity, it is clear that there is still much to be established regarding the effect of LXR signaling on T cells.

While these studies were initially intended to expand upon the dual roles of the RORs and LXRs in T_H_17 cell differentiation and function, we uncovered some novel mechanisms for LXR in CD4^+^ T cell activation and expansion. Not only did LXR activity inhibit the differentiation and function of T_H_1 cells, LXR activity appears to be critical for T_H_1 cell IFNγ production. We demonstrated that GSK2033-mediated LXR inhibition induced a significant, four-fold increase in IFNγ expression whereas GSK2033 had a significantly less effect on IL-4 and IL-17 expression. Additionally, we demonstrated that T09-mediated IL-17 inhibition is due mainly to its activity at the RORs since GSK2033 treatment rescued very little of the T09 mediated IL-17 inhibition in T_H_17 cells. In fact, SR1001 inhibited IL-17 to a greater extent than GW3965 alone, providing further support for the RORs as dominant mediators in IL-17 expression. Interestingly, maximal IL-17 inhibition was seen with T09, suggesting a synergism between the two NRs for T_H_17 cell differentiation and function. Collectively, these data lend some insight into why T09 treatment in various autoimmune models is so efficacious[9], [10]. Therefore, since T_H_1 and T_H_17 cells are both thought to play pathological roles in the induction and progression of EAE, the suppression of both effector populations with T09 explains the greater effect observed than inhibition of one population alone[9], [10]. These studies demonstrate that compounds with mixed pharmacology may be advantageous in the treatment of autoimmune disorders that stem from multiple aberrant effector populations.

While previous studies detailing the *in vivo* efficacy of T09 in autoimmune models is enticing, significant LXR activation in an organism has been shown to affect both fatty acid and carbohydrate metabolism[45]. T09 induces moderate lipogenesis and increases plasma and hepatic triglyceride levels resulting in fatty livers, effects which are undesirable[46], [47]. With these side effects in mind, a potent LXR synthetic compound, like T09, that not only increases lipogenesis and triglyceride levels as well as inhibiting T cell proliferation and potentially inducing cell death is not an optimal option for the development of therapeutics for autoimmune diseases. However, if we use T09 as a scaffold and attempted to retain the efficacy on RORs while minimizing, but not deleting, the effects of LXR, the deleterious side effects may be diminished. The development of this type of compound would not only serve as a useful tool to examine the dual pharmacology of LXRs and RORs, it could provide insight into the usefulness of using similar compounds with mixed pharmacology as potential therapeutics.

In summary, we have demonstrated that the LXR agonist, T09, not only inhibits T_H_17 cell differentiation and function, it also inhibits the differentiation and function of T_H_1, T_H_2, and iT_reg_ cells *in vitro*. The inhibitory mechanism of T09 appears to be mediated through inhibition of T cell proliferation, which ultimately leads to cell death. Our data strongly suggests that synthetic ligands, which weakly induce LXR activation coupled with inhibition of RORs, could yield promise in the treatment of autoimmune diseases that are caused by multiple aberrant effector arms of the immune system, specifically, T_H_1 and T_H_17 cells. T09 is a very efficacious activator of LXR and this likely drives the strong effect on T cell inhibition. One could envision developing a compound with limited LXR agonist activity while retaining strong ROR inverse agonist activity leading to robust inhibition of T_H_17 cell development with more subtle effects on other T cell lineages, including T_H_1 cells.

## Supporting Information

Figure S1 **T09 increases IL-23 expression but not Mertk in splenocyte cultures.** Real Time PCR analysis of splenocytes cultured under T_H_17 polarizing conditions in the presence of T09 (5 µM) for three days. Data are normalized to GAPDH and DMSO. (n = 3, *p<0.05)
(TIF)

Figure S2 **LXR activity regulates cell cycle progression.** Splenocytes cultured under various CD4^+^ T cell polarizing conditions in the presence of DMSO, T09 (5 µM), GSK2033 (5 µM), or T09 plus GSK2033 (5 µM each) for five days. Cells were stained for DNA content with propidium iodide and analyzed by flow cytometry. (*n = 4*)
(TIF)

Figure S3 **LXR activation induces cell death.** Splenocytes cultured under various CD4^+^ T cell polarizing conditions in the presence of DMSO or T09 (3 µM) for five days. Cells were stained with propidium iodide and Annexin V and analyzed by flow cytometry. (*n = 4*).
(TIF)
