# Supplementary figures and images for: LXR-Mediated Inhibition of CD4+ T Helper Cells
Source: PLoS One. 2012 Sep 28;7(9):e46615. doi: 10.1371/journal.pone.0046615 (PMC3460920; doi:10.1371/journal.pone.0046615)

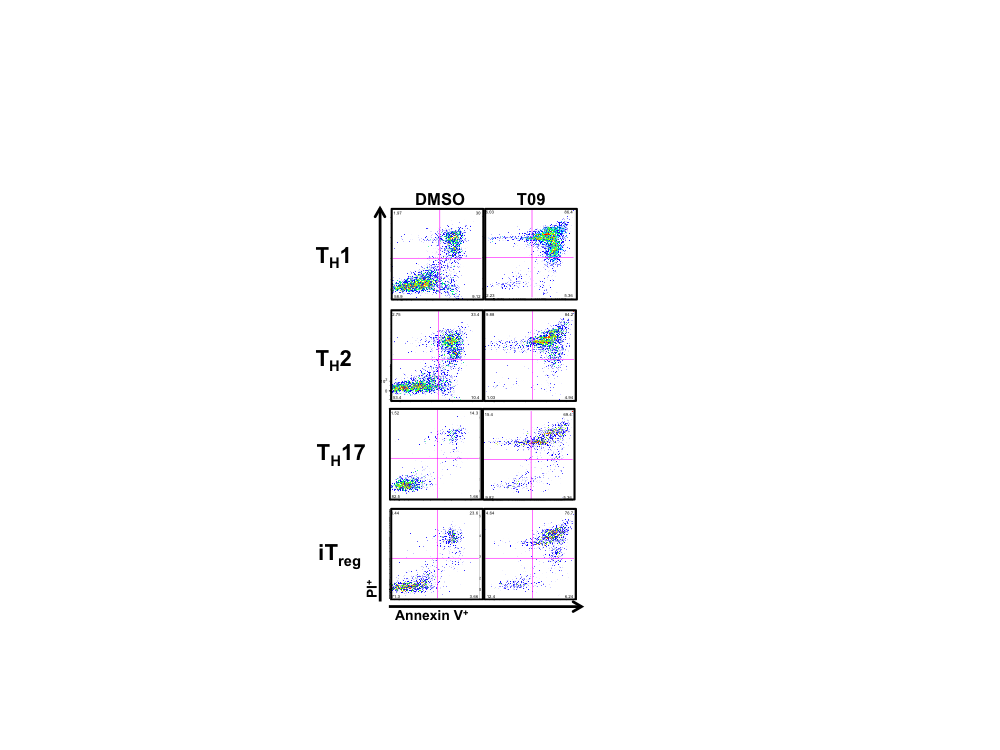

Supplement: Figure S3 — LXR activation induces cell death. Splenocytes cultured under various CD4+ T cell polarizing conditions in the presence of DMSO or T09 (3 µM) for five days. Cells were stained with propidium iodide and Annexin V and analyzed by flow cytometry. (n = 4). (TIF) [file pone.0046615.s003.tif]
